# Phases evolution and photocatalytic activity of Cu_2_O films electrodeposited from a non-pH-adjusted solution

**DOI:** 10.1098/rsos.230247

**Published:** 2023-06-21

**Authors:** Setia Budi, Mari Takahashi, Mega Gladiani Sutrisno, Wisnu Ari Adi, Zahra Fairuza, Budhy Kurniawan, Shinya Maenosono, Akrajas Ali Umar

**Affiliations:** ^1^ Department of Chemistry, Faculty of Mathematics and Natural Sciences, Universitas Negeri Jakarta, Jl. Rawamangun Muka, Jakarta Timur 13220, Indonesia; ^2^ School of Materials Science, Japan Advanced Institute of Science and Technology, 1-1 Asahidai, Nomi, Ishikawa 923-1292, Japan; ^3^ The Centre for Science Innovation, Jakarta 13120, Indonesia; ^4^ Centre for Science and Technology of Advanced Materials, National Nuclear Energy Agency, Kawasan Puspiptek Serpong, Tangerang Selatan, Banten, Indonesia; ^5^ Department of Physics, Faculty of Mathematics and Natural Sciences, Universitas Indonesia, Depok 16424, Indonesia; ^6^ Institute of Microengineering dan Nanoelectronics, Universiti Kebangsaan Malaysia, Selangor 43600, Malaysia

**Keywords:** cuprous oxide, photocatalytic activity, phases evolution, photodegradation, methylene blue, electrodeposition

## Abstract

A pure-phase Cu_2_O film photocatalyst was successfully prepared by the electrodeposition technique from a non-pH-adjusted solution. To investigate the phase evolution and photocatalytic activity of the film, the electrodeposition was conducted at different deposition temperatures. Photocatalytic activity of the films was evaluated from methylene blue (MB) dye degradation. The Cu_2_O phase initially appeared at room temperature and its fraction was found to increase with increasing the deposition temperature, while the impurity phase was successfully diminished. A pure Cu_2_O film with a narrow optical bandgap energy of 1.96 eV was obtained at 75°C. The multi-faceted crystals were found to form at 45°C and became a truncated octahedral structure that possessed {111} and {100} facets as deposition temperature further increased. A preferred orientation growth of {110} facet, which is known to possess a relatively high surface energy, was produced at 75°C. The performance of MB photodegradation enhanced gradually by increasing the deposition temperature. The increase of photocatalytic activity could be attributed to the rise of photoelectrochemical response and the decrease of resistance charge transfer because of narrowing bandgap energy, increasing Cu_2_O fraction, and growing a relatively high catalytic activity facet which had escalated redox reaction that decomposed MB at the photocatalyst–solution interface.

## 1. Introduction

Copper(I) oxide (Cu_2_O) is a p-type semiconductor with a narrow direct bandgap energy of 2.2 eV which is beneficial to the photocatalysed redox reaction under solar radiation [1,2]. Therefore, Cu_2_O is known to be suitable for using the visible light compared with other oxide materials including TiO_2_ [3], ZnO [4], WO_3_ [5], ZnS, [6] ZrO [7] and NiO [8] that have larger bandgap energy in the range of 2.8–4.0 eV, which is more appropriate for ultraviolet (UV) absorption [6]. Whereas sunlight has the main radiation in the range of visible light (47%) and infrared (46%), UV radiation is only 4–7% [9]. Therefore, Cu_2_O has been widely considered to be a promising photocatalyst for the photodegradation of synthetic dyes, which are generally toxic and non-biodegradable. Cu_2_O can be synthesized by various methods such as physical vapour deposition [10], thermal evaporation [11], thermal oxidation of Cu [12], solvothermal [13], plasma-assisted molecular beam epitaxy [14], magnetron sputtering [15], sono-chemical [16], laser ablation [17], pulse laser deposition [18] and spray pyrolysis [19], However, those methods are not cost-effective since the synthesis was conducted under high temperatures from 200°C to 500°C, and, in certain case, under low pressure conditions [20]. By using electrodeposition technique, Cu_2_O could be prepared under relatively low temperature [21–23]. In addition, their microstructure [24], optical [24–26] and photocatalytic properties [24,27] could be easily modified by adjusting the deposition parameters, such as the applied voltage [26] or current [28], and solution pH [24]. Nevertheless, to obtain a phase-pure Cu_2_O, it has been still necessary to study the optimization of deposition conditions. The presence of impurity phases, for example metallic Cu, would degrade photocatalytic performance since it can capture photogenerated electron reducing charge carrier transfer effectivity [10], while other oxides including CuO and Cu_4_O_3_ exhibit such narrow bandgap energy [29], which is favourable for electron–hole pairs recombination that exacerbates their catalytic activities.

In electrodeposition technique, the deposition temperature is known to be one of the most critical parameters to determine the fraction of Cu_2_O phase. Previous studies show the Cu_2_O phase could be formed in the bath temperature range of 25–75°C [25,30–36]. In addition to temperature control, precise adjustment of the pH of the electrodeposition solution was essential to obtain a phase-pure Cu_2_O [25,33,37]. However, since the pH of the solution can change during the reaction, pH control has been extremely difficult. Moreover, the formation of Cu_2_O phase generally requires a relatively long deposition time, which makes pH control even more difficult.

In this study, a non-pH-adjusted solution was used for electrodeposition of phase-pure Cu_2_O photocatalyst. The effect of bath temperature on structure, optical properties and photoelectrochemical responses are investigated. Photocatalytic activity was evaluated from methylene blue (MB) degradation, which is non-biodegradable and toxic, which could cause health problems [38].

## 2. Material and methods

### 2.1. Materials

Analytical grade of copper sulfate pentahydrate (Cu_2_SO_4_ · 5H_2_O) and sodium sulfate (Na_2_SO_4_) were purchased from PT. Merck Tbk, Indonesia. Indium tin oxide (ITO)-coated polyethylene terephthalate (PET) film with sheet resistance of 10 Ω/square was supplied by Kintec Company, Hong Kong.

### 2.2. Electrodeposition of Cu_2_O films

Cu_2_O samples were prepared by electrodeposition from an electrolyte containing 25 mM Cu_2_SO_4_ · 5H_2_O and 0.2 M Na_2_SO_4_ with the solution pH of 5.2. The electrodeposition was conducted in 25 ml of the electrolyte using a three-electrode system with platinum (Pt) wire and Ag/AgCl as counter and reference electrodes, respectively. The ITO with deposition area of 10 × 16.5 mm^2^ was used as a working electrode. The deposition current was fixed at −1 mA using an ER466 potentiostat. The bath temperatures were varied in the range of 10–75°C. The electrodeposited Cu_2_O film was rinsed by distilled water and dried at a room temperature for 2 h. To understand the electrochemical behaviour of the electrolyte system, linear sweep voltammetry (LSV) and cyclic voltammetry (CV) measurements were conducted in the potential range of −0.75 to 1.00 V versus Ag/AgCl with a scan rate of 50 mV s^−1^.

### 2.3. Structural and optical characterization of Cu_2_O films

Crystalline phase analysis of the electrodeposited films was performed by a PANalytical Empyrean X-ray diffractometer (XRD) with Cu K*α* radiation (1.5406 Å). The XRD patterns were analysed using the general structure analysis system (GSAS) to evaluate the structural features and phase composition of the films. X-ray photoelectron spectroscopy (XPS) analysis of the films was carried out on a Shimadzu Kratos AXIS-ULTRA delay-line detector high-performance XPS system with monochromated Al K*α* radiation. The shift of the binding energy scale due to charging was corrected by setting the C 1s peak at 284.5 eV. The core-level XPS spectra were deconvoluted using XPSPEAK software (version 4.1). Morphology of the films was examined by a Fei Inspect F50 field emission scanning electron microscope (FESEM). Optical property was determined by measuring reflectance using a Perkin Elmer LAMBDA 1050 UV–Visible spectrophotometer (UV–Vis) equipped with a diffuse reflectance sphere (DRS).

### 2.4. Photoelectrochemical analysis

A quartz three-electrode cell with Pt wire as a counter electrode and Ag/AgCl as a reference electrode was used for the photoelectrochemical measurements. The electrodes were immersed in a 1.0 M Na_2_SO_4_ electrolyte. All the measurements were carried out under irradiation by a home-made solar simulator equipped with a halogen lamp with the calibrated output intensity of 1 sun (AM 1.5). The electrochemical impedance spectroscopy measurement was performed using a Corrtest CS310 electrochemical workstation in the frequency range of 0.01 Hz–1 kHz. Photoelectrochemical responses were analysed using a linear sweep voltammetry (LSV) method. The photocurrent was recorded in the potential range of −0.5 to 2.0 V versus Ag/AgCl and scan rate of 10 mV s^−1^ controlled by the potentiostat.

### 2.5. Photocatalytic activity measurements

Photocatalytic activity was evaluated based on the degradation of MB measured under irradiation by the solar simulator with intensity of 1 sun (AM 1.5). The MB degradation was determined by recording absorbance at the wavelength of 664 nm using UV–Vis. The photocatalytic activity for MB degradation was measured by the following equation:

2.1 $$\text{\%}\;\mathrm{degradation}=\frac{C_{0}-C_{t}}{C_{0}}\times 100,$$

where *C*_0_ is the initial concentration of MB and *C_t_* is the concentration of MB after *t* s from the start of light irradiation.

## 3. Results and discussion

### 3.1. Structure and phase analyses

**Figure 1.**
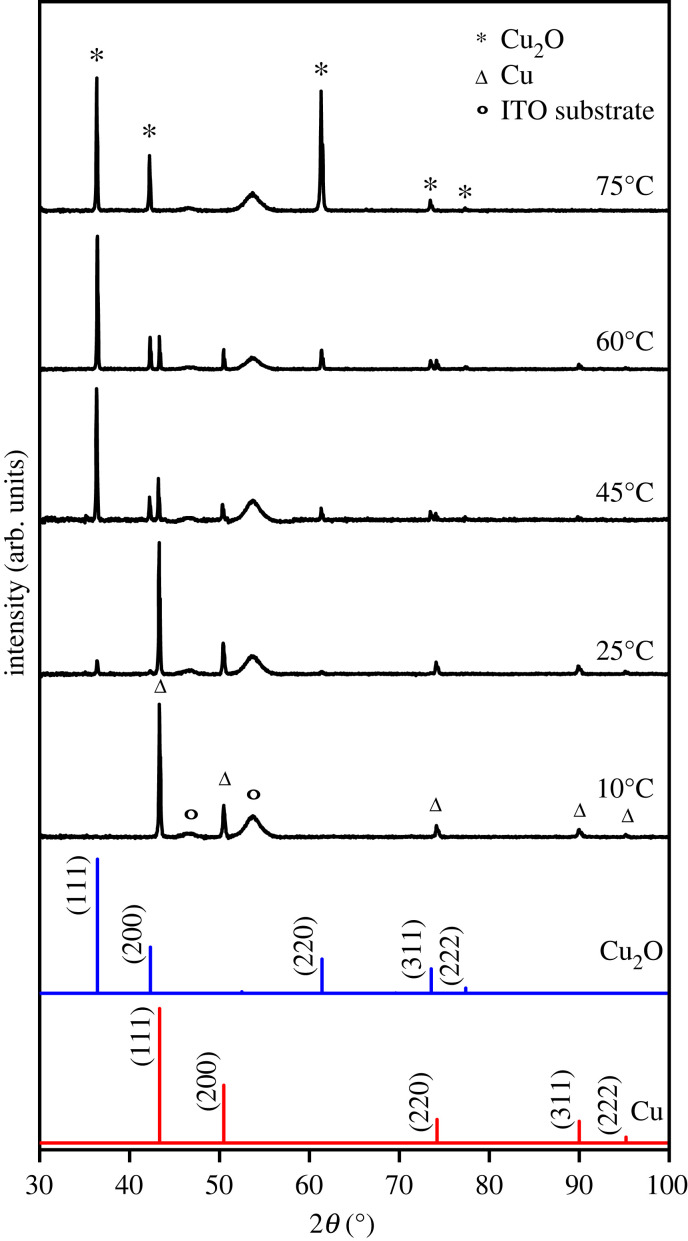
XRD patterns of the electrodeposited films fabricated at different bath temperatures.

**Figure 2.**
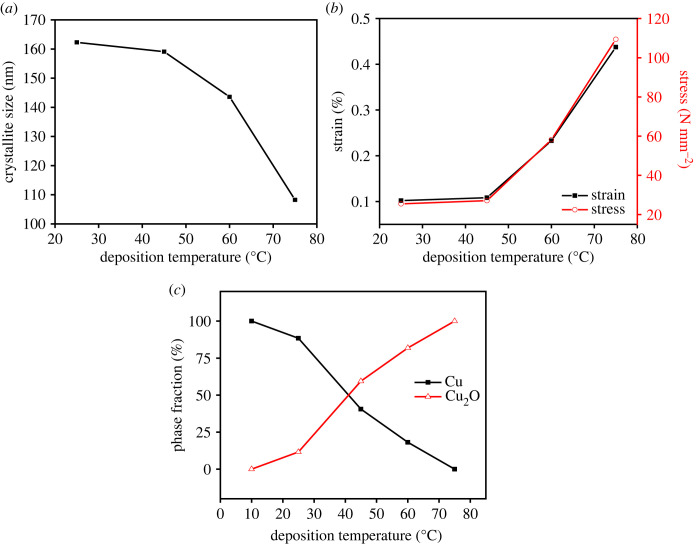
Crystallite size of Cu_2_O (*a*), phase composition (*b*) and strain and stress (*c*) of the electrodeposited films calculated by GSAS analysis.

Figure 1 shows the XRD patterns of the electrodeposited films with different bath temperatures. The Cu_2_O peaks were observed at 2*θ* = 36.33°, 42.21°, 61.29°, 73.44° and 77.28° corresponding to the (111), (200), (220), (311) and (222) planes of cubic Cu_2_O (ICDD no. 01-071-3645), respectively. Meanwhile, the metallic Cu peaks were also observed at 2*θ* = 43.31°, 50.46°, 74.14°, 89.94° and 95.14° assigned to the (111), (200), (220), (311) and (222) planes of cubic Cu, respectively (ICCD no. 01-070-3039). The analysis of XRD patterns using GSAS exhibited that both Cu and Cu_2_O phases are cubic with the space groups of Pn-3m and Fm-3m, respectively. Crystallite sizes of the Cu_2_O were found to decrease at high temperature (figure 2*a*), which could be attributed to an increase of nucleation rate that produced fine grains. This indicates that, in the system, the deposit growth was governed by kinetic factor rather than thermodynamics, which is known to lead the growth of crystal with the increase of temperature. The decrease of crystallite size resulted an increase of strain and stress of the Cu_2_O as shown in figure 2*b*. Based on the XRD patterns, the film prepared at 10°C contained Cu phase only. The Cu_2_O phase started to appear when the deposition was carried out at room temperature. At 75°C, the Cu phase completely disappeared and a single-phase Cu_2_O film was successfully obtained.

**Figure 3.**
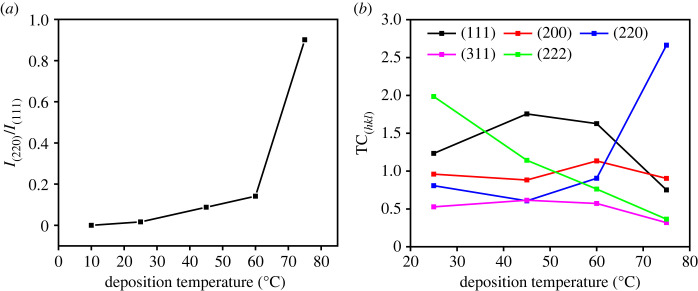
Intensity ratio of I_(220)_/I_(111)_ (*a*) and TC_(hkl)_ (*b*) of the Cu_2_O phase plotted as a function of the bath temperature.

The high diffraction intensity of the (220) indicates a preferred orientation growth of the plane which could be associated with an increase of the growth rate along (111) in the high deposition temperature that widen the {110} facet as shown by the FESEM micrograph (figure 7*e*). Figure 3 shows the high intensity ratio of *I*_(220)_/*I*_(111)_ and texture coefficient, TC_(*hkl*)_, which was measured by equation (3.1) [39], which confirmed the preferential orientation of (220) plane at 75°C.

3.1 $${\mathrm{TC}}_{\left(hkl\right)}=\frac{I_{\left(hkl\right)}/I_{o}\left(hkl\right)}{1/n\sum_{n}I_{\left(hkl\right)}/I_{o}\left(hkl\right)},$$

where *I*_(*hkl*)_ is the plane relative intensity of the measured pattern, *I*_0(*hkl*)_ is the standard intensity of the corresponding plane of the Cu_2_O reference pattern (ICDD no. 01-071-3645), and *n* is the number of reflections. To further confirm the phase evolution, the GSAS evaluation was performed to determine phase composition of the films. The result revealed that the fraction of the Cu_2_O phase gradually increased with the deposition temperature and the phase-pure Cu_2_O film was obtained at 75°C (figure 2*c*). This result shows that a pure Cu_2_O film was successfully prepared from a non-pH-adjusted solution, whereas in previous studies it was necessary to be adjusted to alkaline condition [40,41]. The cathodic reactions of Cu^2+^ are pH dependent. The alkaline condition is required to enable the formation of Cu_2_O through the following reaction:

3.2 $$2{\mathrm{Cu}}^{2+}+2e^{-}+2{\mathrm{OH}}^{-}\to {\mathrm{Cu}}_{2}O+H_{2}O.$$

Under the acidic condition, however, possible reactions are as follow [42]:

3.3 $${\mathrm{Cu}}^{2+}+2e^{-}\to \mathrm{Cu},$$

3.4 $$2{\mathrm{Cu}}^{2+}+2e^{-}+H_{2}O\to {\mathrm{Cu}}_{2}O+2H^{+}$$

3.5 $$\mathrm{and}\;{\mathrm{Cu}}_{2}O+2e^{-}+2H^{+}\to 2\mathrm{Cu}+H_{2}O.$$

Based on equation (3.5), Cu_2_O can be further reduced to Cu, and thus it is difficult to produce phase-pure Cu_2_O in the acidic condition. Nevertheless, in the present study, phase-pure Cu_2_O could be obtained in an acidic solution simply by increasing the deposition temperature.

**Figure 4.**
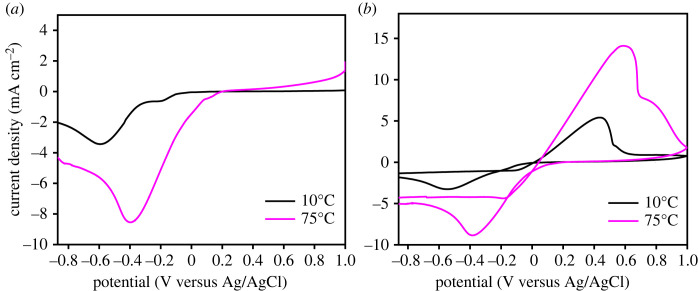
LSV (*a*) and CV (*b*) curves recorded with scan rate of 50 mV s^−1^ at the bath temperatures of 10°C and 75°C.

To investigate the reaction mechanism in the system, cathodic polarization was recorded both at 10°C and 75°C using LSV technique. Figure 4*a* shows that a reduction peak indicating the deposition of Cu_2_O at −0.41 V versus Ag/AgCl was observed at 75°C, while the deposition of Cu was found to occur at 10°C as indicated by the peak at −0.60 V versus Ag/AgCl. To confirm this result, the CV were measured for the same conditions. As a result, an anodic peak corresponding to Cu_2_O oxidation appeared at 0.56 V versus Ag/AgCl at 75°C as shown in figure 4*b*. On the other hand, at 10°C, an anodic peak of Cu oxidation appeared at 0.43 V versus Ag/AgCl (figure 4*b*). It is known that the deposition of Cu_2_O involves oxygen, which is generated by an oxidation reaction at the anode while the Cu^2+^ receives electron at the cathode [42]. In the electrolyte system, ions conductivity and viscosity of the solution is affected by thermal energy [43]. Therefore, the increase in the deposition temperature would promote the generation of oxygen and its mobility, which enhanced the formation of the Cu_2_O as indicated by the increase of deposition rate presented in table 1. The relatively high cathodic current observed at 75°C confirmed a high charge transfer kinetic that showed a higher cathodic reaction rate compared with that of 10°C. Furthermore, the disappearance of the reduction peak to metallic Cu at −0.6 V versus Ag/AgCl, which was observed at 10°C (figure 4*a*), indicates that the reduction process led to Cu_2_O at 75°C. So that the Cu_2_O was successfully electrodeposited from the non-pH-adjusted solution.

**Table 1.** Mass and deposition rate of Cu_2_O, and ratio of Cu in the Cu_2_O to Cu^2+^ in the solution.

bath temperature (°C)	deposit mass (mg)	Cu_2_O fraction (%)	Cu_2_O mass (mg)	Cu_2_O deposition rate (mg cm^−2^ h^−1^)	Cu in the Cu_2_O/Cu^2+^
10	3.20	0	0	0	0
25	6.40	11.59	0.74	0.89	0.016
45	6.50	59.44	3.86	4.68	0.086
60	11.50	81.87	9.41	11.41	0.209
75	13.90	100	13.90	16.85	0.308

### 3.2. X-ray photoelectron spectroscopy analysis and surface morphology

**Figure 5.**
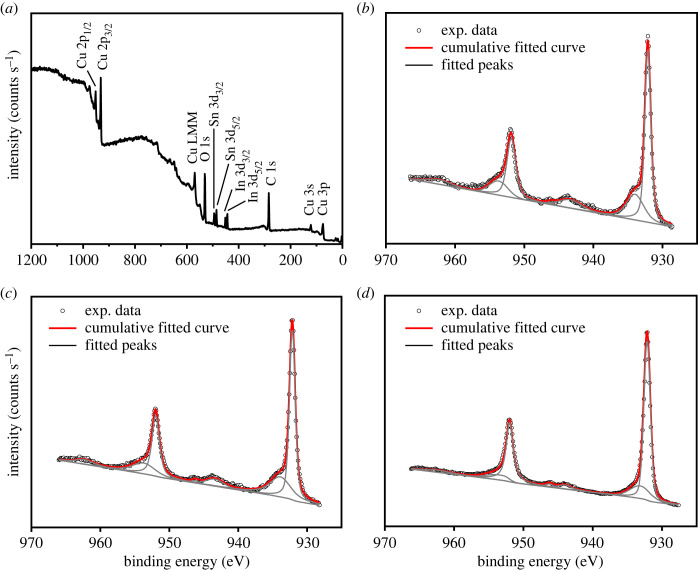
XPS survey spectrum of the film prepared at 45°C (*a*). Cu 2p core level XPS spectra of the films prepared at bath temperature of 45° (*b*), 60°C (*c*), and 75°C (*d*). Open circles, grey curves and red curves represent raw data, deconvoluted peaks (including Shirley background), and the sum of all components, respectively.

To examine the chemical state over the films surface, XPS analysis was carried out for the electrodeposited films with different bath temperatures (45−75°C). Figure 5*a* shows the general survey spectrum of the film obtained at 45°C. The identical spectra, which are not shown in the figure, were observed from other temperatures. There is no other element except Cu, In, Sn, C and O indicating that no impurity element exists in the film. The Cu oxidation state was analysed from the Cu 2p core-level XPS spectra (figure 5*b–d*). The Cu 2p spectra could be deconvoluted into seven peaks with binding energies approximately at 932.1, 933.9, 943.3, 946.1, 951.9, 953.7 and 962.5 eV as shown in figure 5*b–d*.

**Figure 6.**
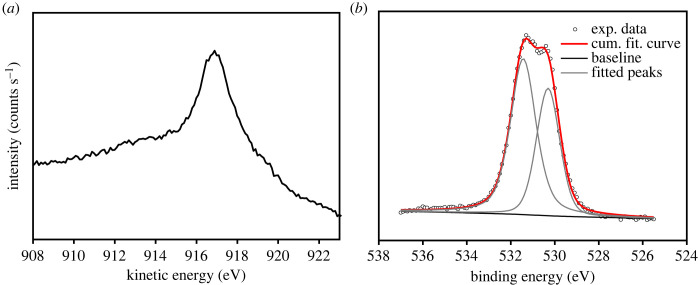
Cu LMM Auger spectrum (*a*) and O 1s spectrum (*b*) of the film prepared at bath temperature of 45°C.

The high-intensity peak at binding energies of 932.1 eV and 951.9 eV can be assigned to the spin-orbit pair of Cu 2p_3/2_ and Cu 2p_1/2_ of a monovalent copper (Cu^+^), respectively. However, it is known to appear very close to the Cu 2p_3/2_ of a metallic copper (Cu^0^) [44]. To distinguish between Cu^+^ and Cu^0^, the Cu LMM Auger spectrum was also recorded. Figure 6*a* shows the Cu LMM Auger spectrum for the film prepared at bath temperature of 45°C as an example. The Cu LMM peak was observed at kinetic energy of 916.9 eV which is characteristic of Cu^+^, while the Cu LMM peak of Cu^0^ is known to appear at 918.6 eV [44]. Therefore, we concluded that the main phase is Cu_2_O and almost no metallic Cu exists on the film surface for all three samples, even though the peak of metallic Cu appears weak in the XRD pattern for the films prepared at 45°C and 60°C (figure 1). The relatively weak peaks at 933.9 and 953.7 eV were assigned to the spin-orbit pair of Cu 2p_3/2_ and Cu 2p_1/2_ of a divalent copper (Cu^2+^), respectively, indicating the presence of a small amount of CuO phase. Since this phase was not observed from the XRD measurements with the penetration depth of up to 20 µm, it could be assumed that CuO only exists on the film surface as a result of a surface-sensitive analysis technique of the XPS. The appearance of weak shake-up satellite peaks of Cu 2p_3/2_ at 943.3 and 946.1 eV and satellite peak of Cu 2p_1/2_ at 962.5 eV, which are known as characteristics of partially filled d orbital (d^9^) of Cu^2+^, confirmed existence of CuO. Therefore, it is also concluded that the surface of the Cu_2_O films was slightly oxidized to CuO. In addition, the fraction of CuO decreased with increasing the deposition temperature, as seen in figure 5*b–d*.

**Figure 7.**
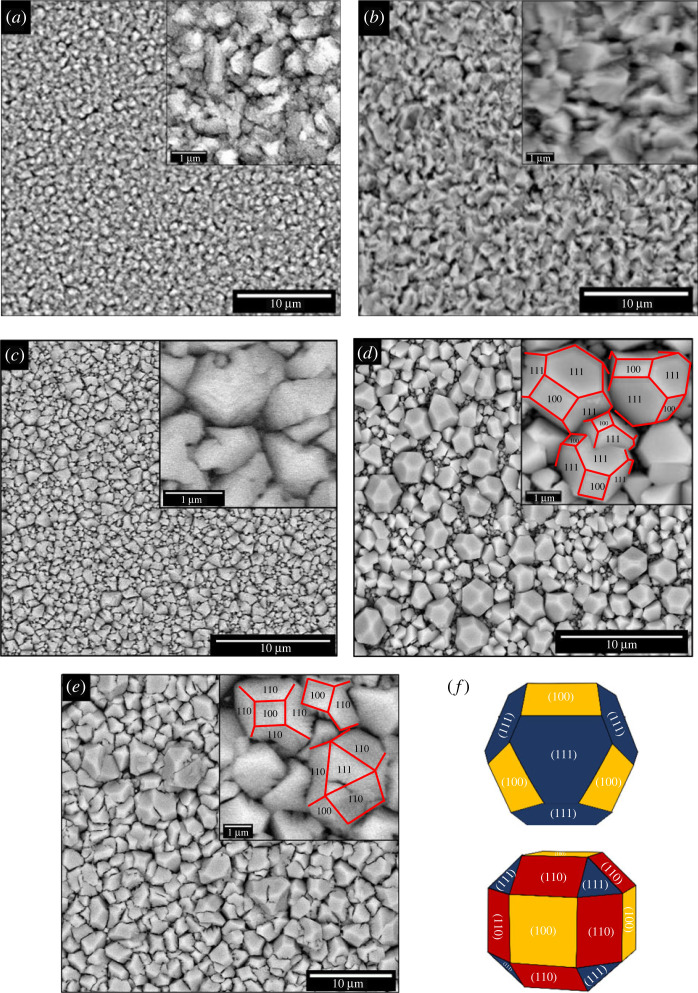
FESEM micrographs of the films prepared at 10°C (*a*), 25°C (*b*), 45°C (*c*), 60°C (*d*) and 75°C (*e*), geometrical configuration of the electrodeposited Cu_2_O crystals exposed {100}, {111} and {110} facets. The inset images are the magnified views of each micrograph.

Morphological features of the films were examined using FESEM as shown in figure 7. From the FESEM micrographs, it is clearly seen that the surface morphology of the film significantly changes depending on the deposition temperature. As the deposition temperature increases, the grain size increases, and the facets appeared clearly. As can be seen from figure 7*c–e*, multi-faceted crystals have started to appear when the deposition temperature was higher than 45°C, which would be due to the growth of the Cu_2_O phase (figure 3*a*). At 60°C, a 14-facet truncated octahedral structure with the facet-index of {100} and {111} was formed (figure 7*d*). When the temperature was increased to 75°C, the Cu_2_O crystal with relatively large {110} facet was observed (figure 7*e*), which is in agreement with the XRD pattern showing a preferred orientation of (220) plane, as shown in figure 1. This morphological change indicates that the growth rate along [111] direction become dominant at 75°C, which then widen {110} plane. Since {110} facet is known to have higher surface energy than those of {100} and {111} facets, it could be assumed that the growth of the exposed facet of the Cu_2_O crystal was kinetically controlled rather than thermodynamically, which is favoured to minimize surface energy.

### 3.3. Optical property

**Figure 8.**
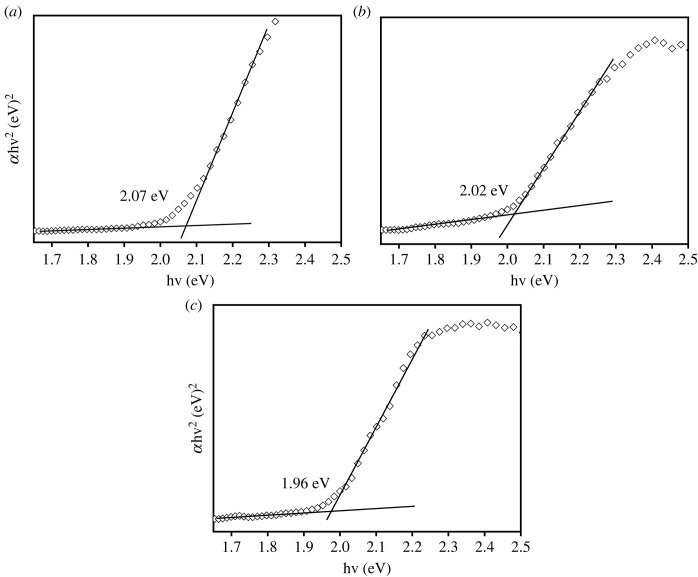
Tauc plots of the films prepared at 45°C (*a*), 60°C (*b*) and 75°C (*c*).

It has been well known that Cu_2_O is a direct bandgap p-type semiconductor with *E*_g_ = 2.17 eV (at 0 K) [45]. To evaluate the bandgap energy (*E*_g_), reflectance spectra of the Cu_2_O-containing films were recorded using UV–Vis equipped with DRS. The *E*_g_ was then determined from the Tauc plot as shown in figure 8. As a result, it was revealed that the phase-pure Cu_2_O film obtained at deposition temperature of 75°C had narrowest *E*_g_ = 1.96 eV, while other films obtained at deposition temperatures of 45°C and 60°C exhibited larger *E*_g_ of 2.07 and 2.02 eV, respectively (figure 8*a,b*). The decrease in *E*_g_ with increasing the deposition temperature is presumably due to the increase of strain and internal stress of the Cu_2_O crystal, as shown in figure 3*b* [46,47].

### 3.4. Photoelectrochemical analysis

**Figure 9.**
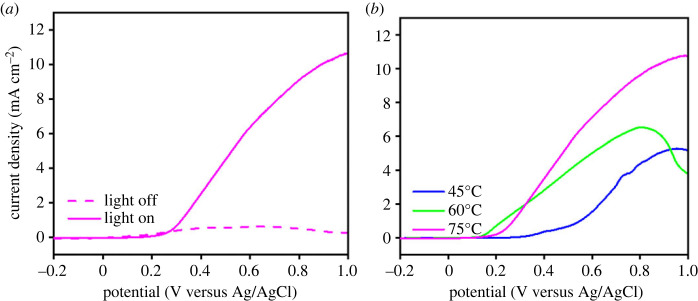
(*a*) Photocurrent recorded with and without solar irradiation from the film prepared at 75°C. (*b*) Photocurrent of the Cu_2_O-containing films recorded under solar irradiation.

To evaluate photoelectrochemical properties of the electrodeposited Cu_2_O, photocurrent was measured by linear sweep voltammetry technique. Figure 9*a* shows that a high photocurrent was observed under solar radiation for the phase-pure Cu_2_O film. On the other hand, almost no dark current was recorded in the swept voltage area. Under radiation, the incoming photons (*hv*) hit the film that generates electrons (e^−^) and holes (h^+^). The photogenerated electrons travel through the system producing photocurrent.

**Figure 10.**
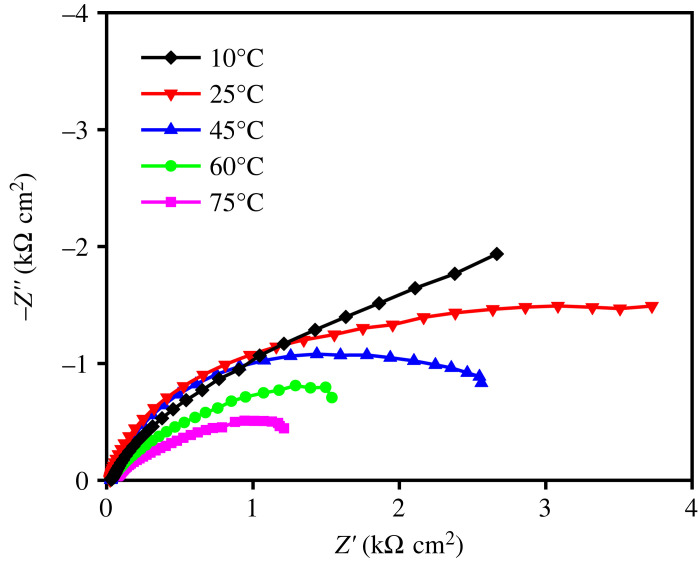
Nyquist plot of the films prepared at different deposition temperatures.

The charge carrier characteristic at the interface between Cu_2_O-containing film and electrolyte was also evaluated from electrochemical impedance spectroscopy (EIS) measurements. Figure 10 presents Nyquist plots showing imaginary part (−Z') as a function of real part (Z') measured under solar irradiation. The arc radius of the plot represents the charge transfer (*R*_ct_) at the electrode–electrolyte interface, where the lower *R*_ct_ designated by the smaller arc radius. From the Nyquist plots, the *R*_ct_ of the film gradually reduced with the increase of the deposition temperature, which could be attributed to the increase of photoexcited electron as charge carrier under radiation. The phase-pure Cu_2_O film deposited at 75°C exhibited the lowest *R*_ct_, indicating a least resistance charge transfer that allow the photoexcited electron to rapidly migrate to the surface and initiate reduction and oxidation reaction at the interface. This conclusion was confirmed by the higher photocurrent that was obtained when the deposition temperature became higher (figure 9*b*). The increase of photocurrent is due to the lessening Cu and increasing Cu_2_O fraction with smaller crystallite size at higher temperature (figure 2*a*). In this case, Cu phase is known to deteriorate photoactivity of the photocatalyst [48], whereas Cu_2_O is responsible for generating charge carrier under solar irradiation. Additionally, the decrease in *E*_g_ that enhances the photon absorption would be one of the reasons for the photocurrent enhancement.

### 3.5. Photocatalytic activity

**Figure 11.**
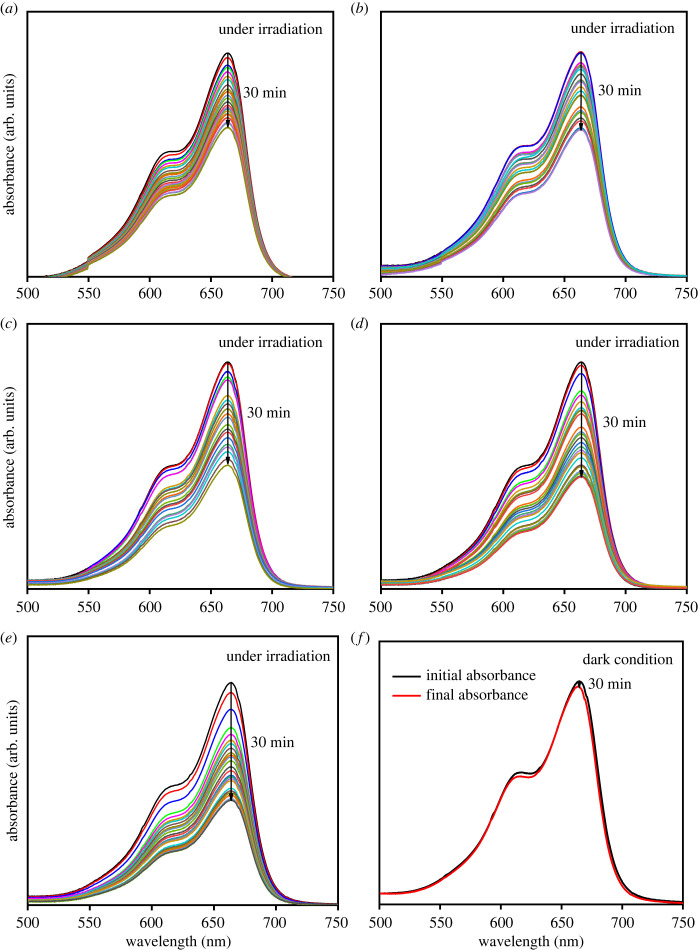
UV–Vis spectra of MB photodegradation performed under irradiation using Cu_2_O prepared at temperature of 10°C (*a*), 25°C (*b*), 45°C (*c*), 60°C (*d*) and 75°C (*e*), and the spectra obtained from dark condition (*f*).

The photocatalytic activity of the films was evaluated from MB degradation under solar irradiation, as shown in figure 11. The absorption peak of MB was observed at a wavelength of 664 nm, and the decrease of the absorption peak intensity corresponds to the degradation of the dye. Under irradiation, the photoexcited electron generates superoxide anion ($\cdot O_{2}^{-}$), while the hole produces hydroxyl radical (·OH). Those free radicals effectively degrade MB, which were adsorbed on the surface of Cu_2_O as indicated by the decrease of absorption peak in the dark condition (figure 11*f*). The formation of radicals can be written in the following reactions:

3.6 $${\mathrm{Cu}}_{2}O+h\nu \to e^{-}+h^{+},$$

3.7 $$e^{-}+O_{2}\to \cdot O_{2}^{-},$$

3.8 $$h^{+}+{\mathrm{OH}}^{-}\to \cdot \mathrm{OH}$$

3.9 $$\mathrm{and}\;h^{+}+H_{2}O\to H^{+}+\cdot \mathrm{OH}.$$

**Figure 12.**
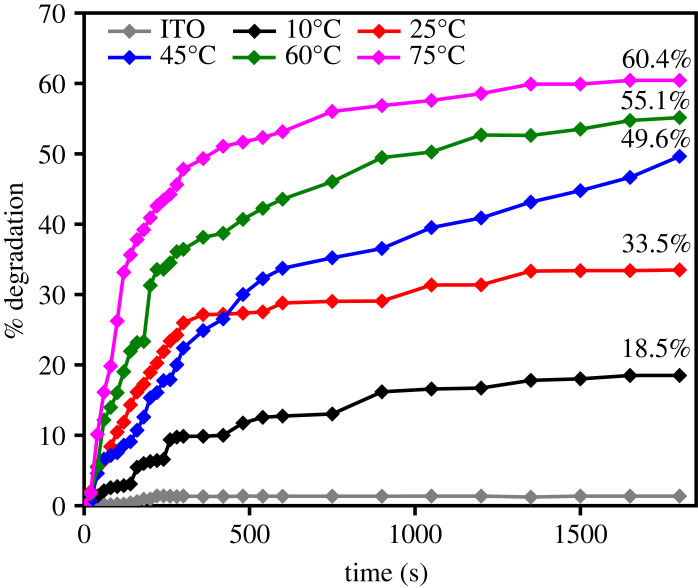
MB Photodegradation using Cu_2_O films prepared at different temperatures.

Based on the spectra, the shrinkage of absorbance intensity was observed to occur rapidly when the deposition temperatures of the photocatalyst increased, which indicates the increase of MB degradation (figure 12). This enhancement of photocatalytic activity is attributed to the phase evolution and morphological features of the films. The high fraction of Cu_2_O that produced a relatively narrow bandgap photocatalyst enhanced photon absorption in the visible light region, which then promoted the formation of photoexcited electrons and holes that increased charge carrier concentration, which was confirmed by the high photocurrent (figure 9), which is responsible for initiating the catalytic reaction with the adsorbed species on the photocatalyst surface. The film morphology is also believed to affect photocatalytic activity in MB degradation. In this case, the morphological changes that led to the formation of multi-faceted structure at 60°C was found to increase photocatalytic activity due to the eight (111) facets [49–52]. As a non-polar surface, (111) facet of Cu_2_O has higher photocatalytic activity compared with that of (100) which is electrically neutral [53]. Previous study has shown that the order of surface energies (*γ*) of the Cu_2_O facets is *γ*_{100}_ < *γ*_{111}_ < *γ*_{110}_ [54]. Therefore, the emerging {110} facet at deposition temperature of 75°C (figure 7*e*) produced a highest photocatalytic activity toward MB degradation.

## 4. Conclusion

Cu_2_O photocatalyst film was prepared from a non-pH-adjusted solution using an electrodeposition technique. The formation of the Cu_2_O in the system is believed to be due to the increase of oxygen generation and its mobility when deposition temperature increased, which led the reduction pathway of Cu^2+^ to produce Cu_2_O. The Cu_2_O fraction in the film increased when temperature varied from 10°C to 75°C showing that the growth of Cu_2_O phase in the system is temperature dependent. The deposition temperature was also successfully employed to modify crystal growth orientation, which produced different exposed facets of the Cu_2_O. The photocatalytic activity test exhibits that MB degradation under solar irradiation increased with the increase of deposition temperature. The enhancement of photocatalytic activity could be attributed to the increase of Cu_2_O fraction, which increased photon absorption in the visible light area and promoted the formation of photoexcited electron–hole pairs, increasing radical production that is responsible for decomposing MB molecule. This conclusion was confirmed by the reduction of charge transfer resistance and the increase of photocurrent under the simulated solar radiation. The structure transformation to multi-faceted crystals that exposes non-polar {111} facet, and a preferred growth orientation of a relatively high surface energy {110} facet of the Cu_2_O phase was also believed to play the role in increasing photocatalytic activity of the film.

## Data Availability

Data are available from the Dryad Digital Repository: https://doi.org/10.5061/dryad.w0vt4b8wr [55].
